# Does diagnostic uncertainty increase antibiotic prescribing in primary care?

**DOI:** 10.1038/s41533-021-00229-9

**Published:** 2021-03-25

**Authors:** Dan Wang, Chaojie Liu, Xinping Zhang, Chenxi Liu

**Affiliations:** 1 School of Medicine and Health Management, Tongji Medical School, Huazhong University of Science and Technology, Wuhan, Hubei China; 2 School of Psychology and Public Health, La Trobe University, Melbourne, VIC Australia

**Keywords:** Drug regulation, Health policy, Health services, Respiratory tract diseases

## Abstract

This study aimed to determine the association between factors relevant to diagnostic uncertainty and physicians’ antibiotic-prescribing behaviour in primary care. A questionnaire survey was conducted on 327 physicians that measured their diagnostic ability, perceived frequency of diagnostic uncertainty, tolerance, and perceived patient tolerance of uncertainty. Physician antibiotic-prescribing behaviours were assessed based on their prescriptions (*n* = 207,804) of three conditions: upper respiratory tract infections (URTIs, antibiotics not recommended), acute tonsillitis (cautious use of antibiotics), and pneumonia (antibiotics recommended). A two-level logistic regression model determined the association between diagnostic uncertainty factors and physician antibiotic prescribing. Physicians perceived a higher frequency of diagnostic uncertainty resulting in higher antibiotic use for URTIs and less antibiotic use for pneumonia. Higher antibiotic use for acute tonsillitis was related to a low tolerance of uncertainty of physicians and patients. This study suggests that reducing diagnostic uncertainty and improving physician and patient uncertainty management could reduce antibiotic use.

## Introduction

Antibiotic resistance is increasingly recognized as a major threat to global health and economic development^1^. There is little doubt that high levels of antibiotic consumption contribute to the development of antibiotic resistance^2^. Over 80% of antibiotics are prescribed in primary care facilities^2^. Irrational antibiotic prescribing in these facilities is common all over the world, including in countries where the antibiotic prescription rate is relatively low. General practitioners in the Netherlands, for example, are relatively less likely to prescribe antibiotics than their counterparts in other countries. However, antibiotic prescribing for patients without an indicative condition remains as high as 59%^3^.

Theoretically, a clear diagnosis is fundamental to guide rational antibiotic prescriptions. The conditions that are most likely to be caused by bacteria are indicative of antibiotic use^4^. Unfortunately, a diagnosis cannot always be established in primary care due to restrictions in resources and diagnostic capacities^5^. The complexity of pathogens adds further challenges to prescribing decisions^6^. Acute tonsillitis, for example, can be caused by viruses or bacteria^7^. A lack of certainty in the diagnosis of illness conditions and pathogens has been blamed for fuelling the over-prescriptions of antibiotics^8–11^.

However, our understanding of how diagnostic uncertainty influences physician use of antibiotics is limited^8–11^, regardless of whether this situation is common in medical practices and is unlikely to disappear in the foreseeable future. Both physicians and patients have to learn to live with uncertainty.

Based on existing research, several factors of physicians relevant to diagnostic uncertainty seemed to play a role contributing to the overuse of antibiotics, including the frequency of diagnostic uncertainty^9^, physician’s diagnostic ability^12^, and physician and patient tolerance of diagnostic uncertainty^13,14^. Existing studies have shown that decrease of physicians’ diagnostic uncertainty has reduced the rates of antibiotic prescription for patients in primary care^9^. Accordingly, it can be expected that where less diagnostic uncertainty takes place, fewer antibiotics are prescribed. It was also shown that nearly half of the irrational use of antibiotics in primary care was due to a deficit in physician’s diagnostic ability^12^. Furthermore, physician and patient tolerance of uncertainty has also been shown to have an impact on clinical decisions^13,14^. These factors may be important when deciding between “tolerance-sensitive” choices^14^: decisions where clinical evidence does not clearly support antibiotic prescription or not and may depend on physician and patient tolerance of uncertainty. However, the effect of these factors on physicians’ antibiotic-prescribing beahaviours has rarely been considered in existing studies.

Thus, this study aimed to determine the association between several factors relevant to the diagnostic uncertainty of physicians and their antibiotic-prescribing behaviours in primary care.

## Results

### Characteristics of antibiotic prescriptions and study participants

**Fig. 1:**
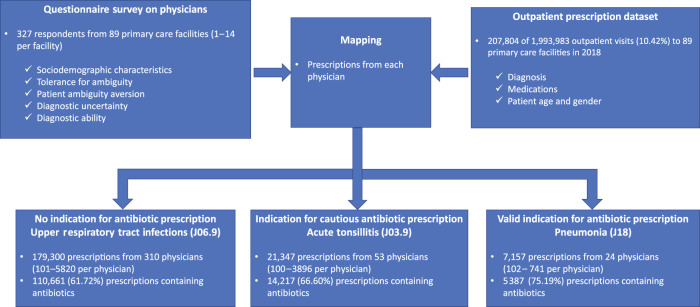
Data collection and extraction procedure. This figure shows the current study design. Physicians’ characteristics regarding diagnostic uncertainty were collected by a questionnaire survey, and their antibiotic-prescribing behaviours in clinical practice were extracted from the outpatient prescription dataset. The surveyed characteristics of physicians were mapped with their prescriptions for three acute conditions commonly treated in primary care facilities, including upper respiratory tract infections, acute tonsillitis, and pneumonia with an unspecified organism. According to the guidelines, antibiotics are not recommended for URTIs, while cautious use of antibiotics can be applied for tonsillitis, and antibiotic prescriptions for pneumonia with an unspecified organism are recommended. These conditions have a varied likelihood of bacterial infection and are deemed sensitive to the physicians and patient tolerance of uncertainty.

The majority of the extracted prescriptions were issued for patients younger than 18 years (35.03%) or between the age of 40 and 64 years (32.96%). Slightly more than half (51.86%) of the patients were male. Approximately 62.68% of the prescriptions contained antibiotics (Fig. 1). Pneumonia was most likely (75.19%) to be prescribed antibiotics, followed by acute tonsillitis (66.60%) and upper respiratory tract infections (URTIs) (61.72%).

The majority of the physician respondents were male (66.97%) and aged between 40 and 59 years (63.3%). Nearly two-thirds (64.22%) were general practitioners. Most respondents (74.62%) had over ten years of clinical experience. The vast majority (81.65%) reported attendance of antibiotic-prescribing training.

### Physician characteristics relevant to diagnostic uncertainty

Approximately 30% of the physician respondents reported a frequency of higher than 40% diagnostic uncertainty in medical practices. The mean score of diagnostic ability rated by the respondents reached 3.36 (SD = 1.14), approximately 67% of the maximum score of five. Less than half (48.93%) obtained a score higher than three. Physicians’ low level of tolerance of uncertainty was found, with the respondents having a mean score of 22.53 (SD = 4.87), 64% of the maximum possible score of 35. The physician respondents perceived a high level of patient tolerance of diagnostic uncertainty, with less than half (45.57%) reporting a score higher than three. The mean score of patient tolerance of uncertainty was 3.82 (SD = 3.60), only 24% of the maximum possible score of 16 (Table 1).

**Table 1:** Antibiotic prescription rates in primary care physicians with different levels of factors relevant to diagnostic uncertainty.

Characteristics	Total			Upper respiratory tract infections			Acute tonsillitis			Pneumonia		
	*N*	(%)	Antibiotic prescription rate (mean ± SD)	*N*	(%)	Antibiotic prescription rate (mean ± SD)	*N*	(%)	Antibiotic prescription rate (mean ± SD)	*N*	(%)	Antibiotic prescription rate (mean ± SD)
Physician perceived frequency of diagnostic uncertainty (range 0–3)												
<2	230	(70.34)	62.01 ± 18.26	218	(70.32)	60.78 ± 18.40	34	(64.15)	75.29 ± 17.86	16	(66.67)	73.68 ± 16.31
≥2	97	(29.66)	62.35 ± 18.82	92	(29.68)	61.16 ± 19.29	19	(35.85)	81.90 ± 14.17	8	(33.33)	74.46 ± 11.99
Physician diagnostic ability (range 0–5)												
≤3	167	(51.07)	64.65 ± 16.76	155	(50.00)	62.64 ± 16.77	28	(52.83)	82.62 ± 12.45	18	(75.00)	72.29 ± 12.28
>3	160	(48.93)	59.47 ± 19.67	155	(50.00)	59.15 ± 20.24	25	(47.17)	72.11 ± 19.39	6	(25.00)	63.88 ± 18.06
Physician tolerance of uncertainty^a^ (range 0–35)												
≤21	139	(42.51)	63.39 ± 17.60	124	(40.00)	61.45 ± 18.10	23	(43.40)	73.51 ± 18.24	16	(66.67)	72.31 ± 14.13
>21	188	(57.49)	61.17 ± 18.96	186	(60.00)	60.52 ± 19.03	30	(56.60)	80.84 ± 15.15	8	(33.33)	77.20 ± 16.39
Patient tolerance of uncertainty^b^ (range 0–16)												
≤3	178	(54.43)	62.00 ± 18.45	165	(53.23)	60.56 ± 19.03	31	(58.49)	76.23 ± 18.49	14	(58.33)	72.43 ± 15.78
>3	149	(45.57)	62.25 ± 28.39	145	(46.77)	61.27 ± 18.24	22	(41.51)	79.67 ± 14.25	10	(41.67)	76.06 ± 13.69

^a^Physician tolerance of uncertainty was assessed using tolerance for ambiguity scale, with higher scores indicating a physician’s lower level of tolerance of uncertainty.

^b^Patient tolerance of uncertainty was assessed based on physician estimation of patient ambiguity aversion, with higher scores indicating a lower level of patient tolerance of uncertainty.

### Effects of diagnostic uncertainty on antibiotic prescribing

The logistic regression models showed that physicians with a higher perceived frequency of diagnostic uncertainty were more likely to prescribe antibiotics to patients with URTIs (adjusted odds ratio (AOR) = 1.034, *p* = 0.004) and less likely to prescribe antibiotics to patients with pneumonia (AOR = 0.575, *p* = 0.001). Physicians with higher diagnostic ability were less likely to prescribe antibiotics for patients with URTIs (AOR = 0.891, *p* < 0.001) and acute tonsillitis (AOR = 0.813, *p* = 0.026). In addition, for patients with acute tonsillitis, every one-unit increase in score of TFA (tolerance for ambiguity) (indicating a lower level of physician tolerance of uncertainty) showed a 9.8% increase in the odds of patients being prescribed with antibiotics (AOR = 1.098, *p* < 0.001) and every one-unit increase in score of patient AAM (Ambiguity Aversion in Medicine) (indicating a lower level of patient tolerance of uncertainty) showed a 34.3% increase in the odds of patients being prescribed with antibiotics (AOR = 1.343, *p* = 0.005), respectively (Table 2).

**Table 2:** Effects of diagnostic uncertainty on antibiotic-prescribing behaviours in primary care physicians.

Characteristics of diagnostic uncertainty	Upper respiratory tract infections		Acute tonsillitis		Pneumonia	
	AOR (95% CI)^a^	*p*	AOR (95% CI)^a^	*p*	AOR (95% CI)^a^	*p*
Physician perceived frequency of diagnostic uncertainty	1.034 (1.011, 1.057)	0.004	0.912 (0.774, 1.076)	0.275	0.575 (0.417, 0.793)	0.001
Physician diagnostic ability	0.891 (0.876, 0.907)	<0.001	0.813 (0.677, 0.975)	0.026	1.056 (0.847, 1.318)	0.627
Physician tolerance of uncertainty	0.998 (0.990, 1.006)	0.610	1.098 (1.043, 1.155)	<0.001	1.071 (0.915, 1.254)	0.393
Patient tolerance of uncertainty	0.989 (0.943, 1.038)	0.659	1.343 (1.091, 1.653)	0.005	0.976 (0.597, 1.597)	0.923
Physician tolerance of uncertainty × Patient tolerance of uncertainty	1.002 (1.000, 1.004)	0.132	0.986 (0.977, 0.995)	0.003	1.000 (0.982, 1.018)	0.979

^a^*AOR* Adjusted odds ratio, odds ratio adjusted for variations in patient demographic characteristics (age and gender) and sociodemographic characteristics of primary care physicians (age, gender, qualification, income, workplace, clinical unit, clinical expertise, professional title, and antibiotic training).

**Fig. 2:**
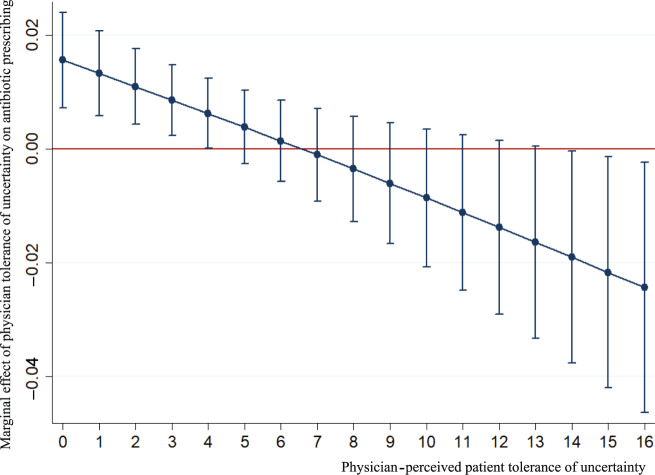
The interaction effect between physician tolerance of uncertainty and patient tolerance of uncertainty on antibiotic prescribing for acute tonsillitis. This figure shows the interaction between physician tolerance of uncertainty and perceived patient tolerance of uncertainty regarding antibiotic use with 95% confidential intervals for outpatients with acute tonsillitis. Physician tolerance of uncertainty was assessed using a tolerance for ambiguity scale, with higher scores indicating lower physician tolerance of uncertainty. Patient tolerance of uncertainty was assessed based on physician estimation of patient ambiguity aversion, with higher scores indicating lower patient tolerance of uncertainty. For different perceived patient tolerances of uncertainty, the one-point decrease in physician tolerance of uncertainty played different roles. When physicians perceived high patient tolerance of uncertainty (scores ≤6), the lower physician tolerance of uncertainty further increased antibiotic use, while for perceived low level of patient tolerance of uncertainty (scores ≥7), the lower physician tolerance of uncertainty would reduce antibiotic use. However, the interaction between the physician tolerance of uncertainty and perceived patient tolerance of uncertainty on antibiotic use was only significant when perceived patient tolerance of uncertainty was ≤4 (marginal effect >0, *p* < 0.05) or ≥14 (marginal effect <0, *p* < 0.05).

In addition, the interaction effect between patient and physician tolerances of uncertainty on antibiotic prescribing for acute tonsillitis was also statistically significant (adjusted odds ratio, AOR = 0.986, *p* = 0.003) (Table 2). To show the detailed effect of interaction between physician and patient tolerance of uncertainty on antibiotic prescribing for acute tonsillitis, the marginal effect of physician tolerance of uncertainty on antibiotic prescribing when physicians interacted with different levels of patient’s tolerance of uncertainty was presented (Fig. 2). When interacted with patients with high tolerance of uncertainty (scores ≤4), physician’s one-unit increase in score of TFA would further increase the odds of antibiotic use (marginal effect >0, *p* < 0.05). In contrast, when interacted with patients with low tolerance of uncertainty (scores ≥14), physician’s one-unit increase in score of TFA would further reduce the odds of antibiotic use (marginal effect <0, *p* < 0.05). However, the marginal effect size remained low (up to 0.024). Combined with the effect of physician tolerance of uncertainty on antibiotic prescribing (Table 2), this indicated that the overall effect of physician’s one-unit increase of tolerance score increased the odds of antibiotic prescribing, ranging from 7.4% to 11.4% based on the different level of patient tolerance of uncertainty.

## Discussion

This study offers some new insight into the antibiotic-prescribing behaviours of primary care physicians. Previous studies have identified insufficient knowledge^15^ and perverse incentives^16^ as the main drivers of the over-prescriptions of antibiotics in primary care physicians. The findings of this study contribute to the advancement of our understanding of such a complicated behavioural issue. The work of primary care physicians usually involves the treatment of illness conditions without a confirmed diagnosis, let alone a clear pathogen origin. Often, they have to make a clinical judgement and decision without further actions to clear the diagnostic uncertainty due to restrictions in time, technologies, and resources.

Our study shows that the physician perceived accumulated frequency of diagnostic uncertainty and diagnostic ability of primary care physicians are associated with antibiotic prescribing practices, albeit to varied degrees across illness conditions. Higher exposure to diagnostic uncertainty is associated with a higher level of antibiotic prescribing for URTIs, a condition where antibiotics are not recommended, but a lower level of antibiotic prescribing for pneumonia, a condition where antibiotics are recommended. In contrast, physicians’ and patients’ low perceived tolerances of uncertainty are associated with antibiotic prescribing for acute tonsillitis, a condition in which antibiotics are optional depending on the clinical judgement of prescribers.

Clearly, diagnostic uncertainty is a significant barrier to implementing evidence-based practice guidelines. However, the certainty of recommendations in clinical guidelines may also influence the way in which the recommendations are adopted. It appears that the effect of physicians’ low tolerance of uncertainty varies with the instructions in the clinical guidelines. Physician low tolerance of uncertainty may have a limited effect, if any, on antibiotic prescribing for the conditions that antibiotics are unambiguously recommended or not recommended.

The significant association between the low tolerance of uncertainty and increased antibiotic prescribing in primary care deserves increased research and policy attention. This study revealed that such an association is significant for acute tonsillitis, a condition that requires profound individual clinical judgement for antibiotic prescribing according to the clinical guidelines. Overall, the established link agrees with the results from several studies in which physician fear of uncertain consequences was found to have fuelled antibiotic prescriptions^3,17,18^. However, this study also demonstrated that low tolerance of uncertainty was not significantly associated with antibiotic prescribing for URTIs or pneumonia, for which individual clinical judgement is virtually discouraged in the clinical guidelines. Esch and colleagues argued that physician and patient attitudes are particularly important in situations where antibiotic prescriptions are not clearly arbitrated in clinical guidelines^3^.

The effect of physicians’ tolerance of uncertainty on antibiotic prescribing is shaped by their perceived patient tolerance of uncertainty according to the significant interaction effect as revealed in this study (see Table 2 and Fig. 2). Previous studies have shown that patient preference has a significant impact on antibiotic-prescribing decisions in general practice^3^. Physicians tend to change strategies in dealing with uncertainty when they perceive different levels of patient tolerance of uncertainty^19^. In the current study, we found that when interacted with different levels of patient tolerance of uncertainty, the effect of physician tolerance of uncertainty can go towards two different directions. Specifically, when interacted with patients with high tolerance of uncertainty, an increase in physicians’ scores in TFA (indicating a decrease of tolerance of uncertainty) would further increase the odds of antibiotic use. In contrast, when interacted with patients with low tolerance of uncertainty, an increase in physicians’ scores in TFA would further reduce the odds of antibiotic use (see Fig. 2). Patients who show a low tolerance of diagnostic uncertainty are likely to have strong cognitive, affective, and behavioural reactions^20^. They are less likely to trust in and follow advice from primary care physicians. Therefore, primary care physicians may choose to refer these patients to a specialist without necessarily prescribing antibiotics. Such a strategy is often considered important to avoid potential patient complaints and even legal disputes in China^21^.

The overuse of antibiotics in primary care is still common in China despite great policy efforts in both restrictive and persuasive interventions. The average antibiotic prescribing rate per physician in this study ranged from 61% to 74% for different conditions, which represents a reduction of 20 percentage points compared to the level in 2009–2011 (ref. ^16^). However, there is still space for further improvement.

The results of this study imply that reducing diagnostic uncertainty may have the potential to improve compliance with clinical guidelines. This is consistent with the findings of an earlier multi-centre clinical trial that used procalcitonin as a biomarker to reduce antibiotic prescribing in primary care^9^. Procalcitonin gives a consistent indication of bacterial infection that can guide antibiotic prescriptions^22^. Higher diagnostic ability is indeed associated with lower levels of irrational antibiotic prescriptions according to the findings of this study, which is also supported by previous studies^12,22^. In general, physicians with high diagnostic ability are less likely to consider antibiotics as a substitute for deficits in diagnoses^23^. However, it is important to note that this study assessed the effect of the physician’s perceived accumulated frequency of diagnostic uncertainty of primary care physicians, which is different from diagnostic uncertainty at the point of care. Further studies are needed to test whether the use of procalcitonin can attenuate the negative effect of past experiences of diagnostic uncertainty on compliance with clinical guidelines for antibiotic prescribing.

High levels of diagnostic uncertainty have been experienced by primary care physicians. Earlier studies have highlighted the importance of reducing diagnostic uncertainty^9,12^. However, the appropriate management of uncertainty is equally, if not more, important in curbing the over-prescription of antibiotics in primary care. Unfortunately, limited studies have addressed this issue^24^. In a study conducted in the US, the physicians participated in a set of small-group, practice-based activities to share clinical experiences and learn how to manage the dilemma of clinical uncertainty^25^. The outcome remains unknown since only qualitative feedback from the physician was assessed. Further studies on effective interventional strategies in relation to the management of uncertainty from both physician and patient perspectives are warranted. Theoretically, the management of uncertainty involves cognitive, emotional, and ethical considerations^20^. A comprehensive interventional programme that can be incorporated into clinical guidelines has yet to be developed.

In addition, it is important to mention that physician and patient tolerance of uncertainty were scored as continuous factors, and, for instance, an AOR of 1.098 means that, for every 1-unit increase in the score, a 9.8% increase in the odds of antibiotic prescribing is expected (see Table 2: Physician tolerance of uncertainty). Considering the effect size and the variation in physician and patient tolerance of uncertainty (physicians: 18–27, patients: 0–7), improving physician and patient management of uncertainty could considerably reduce antibiotic prescribing, at least for acute tonsillitis, for which antibiotics are not clearly recommended.

In this study, the questionnaire survey data at the physician level were linked with the antibiotic prescription dataset at the patient level. The multilevel modelling allowed us to account for the nested structure of the data and determine the associations between several factors relevant to the diagnostic uncertainty of physicians and their prescribing behaviours for various illness conditions of patients. The sample size was large.

Nevertheless, there were several limitations. Patient tolerance of diagnostic uncertainty was estimated based on the perceptions of the physicians in this study. This reflects the overall experience of the physicians, rather than the specific circumstance when the individual prescriptions were issued. The study was conducted in Hubei, a region with a relatively high level of antibiotic prescriptions in China. Caution should be taken in attempts to generalize the findings to other regions or countries.

Primary care physicians commonly face diagnostic uncertainty in clinical practice, and its frequency is associated with their antibiotic prescribing practices. However, these associations vary by illness conditions, with a higher frequency of diagnostic uncertainty resulting in higher antibiotic use for URTIs (antibiotics not recommended) and lower use for pneumonia (antibiotics recommended). For acute tonsillitis, in which antibiotics should be cautiously used depending on the clinical judgement of prescribers, tolerance of uncertainty, rather than the frequency of diagnostic uncertainty, was associated with antibiotic-prescribing behaviours, with lower physician and patient tolerance of uncertainty resulting in a higher use of antibiotics. This study suggests that reducing diagnostic uncertainty as well as better physician and patient uncertainty management could reduce antibiotic use.

## Methods

### Study design

This study adopted a cross-sectional design involving three main stages. First, a questionnaire survey of physicians was conducted to assess their characteristics relevant to diagnostic uncertainty. Second, physicians’ actual antibiotic-prescribing behaviours were evaluated based on their prescription data in three common diagnoses in primary care. Finally, we linked physician survey data with their prescription data to determine the association between factors relevant to diagnostic uncertainty and physician antibiotic-prescribing behaviour (Fig. 1).

The three diagnoses included in the current study are URTIs, acute tonsillitis, and pneumonia with an unspecified organism, which were selected based on the national clinical guidelines for antimicrobial agents in China (2015 version)^26^ and the USA^6^. Because these conditions have a varied likelihood of bacterial infection, antibiotics are not recommended for URTIs, the cautious use of antibiotics is applied for tonsillitis, and antibiotics are recommended for pneumonia. Considering that different diagnoses require varied decision making regarding antibiotic use, prescriptions and their prescribers were classified into three groups based on the three diagnoses. We separately analysed each group to determine the association between factors relevant to diagnostic uncertainty and physician antibiotic-prescribing behaviours.

### Sampling and participants

Two-stage cluster random sampling was adopted for the selection of primary care facilities in Hubei Province, China. In the first stage, the provincial capital, Wuhan, and the other four prefecture-level cities were randomly selected from the 16 cities in Hubei. In the next stage, five urban and five rural districts were further randomly selected in each included city. Within each district, all primary care facilities were included. This process resulted in 89 primary care facilities being included (25 healthcare community centres in urban areas and 64 township hospitals in rural areas), which covered a geographic area with 6.55 million people (approximately 11% of all populations in Hubei)^27^.

According to the recommendation from the World Health Organization for a reliable estimate of physician antibiotic-prescribing patterns, physicians who issued at least 100 prescriptions for any one of the three selected diagnoses were eligible for the questionnaire survey. Based on the inclusion criteria, the sampling process generated a sample size of 469 eligible physicians from all included primary care facilities.

### Data collection

Two steps were involved in data collection. The first step was a self-administered questionnaire survey of factors relevant to diagnostic uncertainty, collecting physician diagnostic ability, perceived frequency of diagnostic uncertainty, physician tolerance of uncertainty, and perceived patient tolerance of uncertainty. The second step was an assessment of the antibiotic-prescribing behaviours of these physicians based on their prescriptions in relation to the three selected illness conditions.

In the first step, trained investigators were paired and deployed to the participating facilities to collect data from November 2019 to January 2020. Before the survey, all investigators had completed 1 day of intensive training, covering the background of the survey, a detailed explanation of the survey instrument, and a simulated survey test. The pairing of investigators aimed to help each investigator when one omitted some required procedures to ensure a standardized survey process.

In total, 469 eligible physicians were invited to participate in this study. A total of 327 (69.72%) physicians returned a valid self-completed questionnaire.

In the second step, prescriptions issued by the 327 questionnaire respondents were extracted from the 2018 outpatient prescription dataset gathered by the local governments. The International Classification of Diseases (ICD-10) codes J06.9 (URTIs), J03.9 (acute tonsillitis), and J18 (pneumonia with unspecified organism) were used to identify prescriptions for the three acute illness conditions. This resulted in a total of 207,804 prescriptions: 179,300 prescriptions for URTIs from 310 physicians; 21,347 prescriptions for acute tonsillitis from 53 physicians; and 7157 prescriptions for pneumonia from 24 physicians (Fig. 1). The extracted prescriptions also recorded patient age and gender.

### Measurements

The use (Yes = 1, No = 0) of antibiotics (recorded as Anatomical Therapeutic and Chemical subgroup J01) in each prescription served as the dependent variable.

The independent variables contained two levels of measurements: prescription and physician levels. The prescription-level measurements included patient age, gender, and illness conditions embedded in the prescriptions. The physician-level measurements were captured by the questionnaire survey, which included sociodemographic characteristics of the physicians (age, gender, qualification, income, workplace, clinical unit and expertise, professional title, antibiotic training), and their perceived frequency of diagnostic uncertainty, diagnostic ability, tolerance of uncertainty, and patient tolerance of uncertainty.

There is still no clear definition of what “diagnostic uncertainty” means and no comprehensive framework for its measurement in medical practice. Thus, we referred to a recent systematic review^28^ and defined diagnostic uncertainty as the “subjective perception of an inability to provide an accurate explanation of the patient’s health problem”. This resulted in the adoption of the common practices in existing studies in which factors relevant to diagnostic uncertainty were assessed based on physicians’ perception or estimation^28^.

Physician perceived frequency of diagnostic uncertainty assessed the frequency with which physicians felt unable to provide a clear diagnosis to their patients. It was measured by one item: “in the past year for patients you prescribed antibiotics, about what percentage were you confident in giving a clear diagnosis?”, which has been validated in a previous study in the primary care setting^5^. Respondents were asked to rate their experience on a five-point scale from 0–20% to 80–100%. Then, the reply was reserve coded with higher scores representing a higher frequency of diagnostic uncertainty (score 0: ≥80% patients were confidently given a clear diagnosis to score 3: <40% patients were confidently given a clear diagnosis).

Diagnostic ability assessed the ability of physicians to correctly diagnose patient diseases based on five scenarios concerning influenza, bronchitis, pneumonia, pyelonephritis, and enteritis. These diseases were selected since they are the common reasons for the irrational use of antibiotics^29^. Respondents were asked to identify one correct answer from five alternative options for each scenario. A summed score for each respondent was calculated, with a higher score indicating higher diagnostic ability.

Physician tolerance of uncertainty assessed physicians’ atttitudes towards uncertainty, which was measured by the seven-item TFA scale^30^. According to the TFA scale, respondents were asked to assess their attitudes towards statements relevant to uncertainty, for example, “before any important task, I must know how long it will take”. A six-point Likert scale was applied, ranging from 0 = “strongly disagree” to 5 = “strongly agree”, and the replies of all items were in the same direction; a higher score indicated a lower tolerance. A summed score for each respondent was calculated, with a higher score indicating a lower tolerance of uncertainty.

Patient tolerance of uncertainty assessed patient attitudes towards diagnostic uncertainty, which was measured using the four-item AAM scale^19^. This scale measures the negative reactions of patients to diagnostic uncertainty, including (i) becoming unwilling to have the test or treatment, (ii) finding the information upsetting, (iii) becoming less trustful of medical experts, and (iv) losing confidence in the test or treatment. Respondents were asked to estimate the percentage of their patients holding negative reactions on a five-point Likert scale, ranging from 0 = “lower than 20%” to 4 = “higher than 80%”. The replies of all items are in the same direction that a higher score indicates a lower level of patient tolerance of uncertainty. A summed score was calculated for each respondent, with a higher score indicating a lower level of patient tolerance of uncertainty.

The above scales were tested in a pilot study of 24 primary care physicians, which demonstrated acceptable internal consistency with Cronbach’s alpha ranging from 0.69 to 0.84.

### Statistical analysis

The characteristics of the participating physicians and their patients for whom the prescriptions were issued were described through frequency analyses.

A multivariate logistic regression model was established for each of the three illness conditions to determine the associations between factors relevant to the diagnostic uncertainty of physicians and their antibiotic-prescribing behaviours in primary care. The models were adjusted for variations in the demographic characteristics of patients (age and gender) and sociodemographic characteristics of physicians (age, gender, qualification, income, workplace, clinical unit, expertise, professional title, and antibiotic training). These control variables were identified as potential predictors of antibiotic prescriptions in previous studies^17,18^.

The regression models involved two levels, as the data were hierarchically structured with prescriptions nested in physicians. The two-level analyses allowed us to take into account the nested structure of the data and the differences in the number of patients per physician. Apart from the main effects of physician perceived frequency of diagnostic uncertainty, diagnostic ability, physician tolerance of uncertainty, and patient tolerance of uncertainty, the interaction effect between physician tolerance and patient tolerance of uncertainty on antibiotic prescribing was also tested in the models. According to existing studies, the main effect evaluates the relationship between independent variables and the dependent variable, while the interaction effect assesses the effect between the independent variables that impacts the dependent variable^19^. In the current study, the interaction term was introduced to the model because the prescribing behaviours of physicians varied with patient tolerance of uncertainty, and prescription decisions were considered a consensus between physicians and patients. To illustrate how the interaction influenced antibiotic prescribing, the plot of the marginal effect of the interaction between the two independent variables (physician tolerance of uncertainty × patient tolerance of uncertainty) on the dependent variable (antibiotic prescribing) was presented^31^.

All analyses were performed using STATA (version 14.0). The level of statistical significance was set at *p* < 0.05.

### Ethics

This study was approved by the Ethics Committee of Tongji Medical College, Huazhong University of Science and Technology (No: 2020-S099). Written consent was obtained before the survey from each participant in the current study.

### Reporting summary

Further information on research design is available in the Nature Research Reporting Summary linked to this article.

## Supplementary information

Supplementary Information

Reporting Summary

## Data Availability

The data of this study are derived from surveyed local institutions and restrictions apply to its availability, which were used under licence for the current study, and so are not publicly available. Data are, however, available from the authors upon reasonable request and with permission of surveyed local institutions and governments.
