# Supplementary material for: Does diagnostic uncertainty increase antibiotic prescribing in primary care?
Source: NPJ Prim Care Respir Med. 2021 Mar 25;31:17. doi: 10.1038/s41533-021-00229-9 (PMC7994848; doi:10.1038/s41533-021-00229-9)
Supplement: Supplementary file 1 — Supplementary Information [file 41533_2021_229_MOESM1_ESM.pdf]

Supplementary Table 1:

Characteristics of included physicians

**Characteristics**	**Diagnosis based on ICD-10**			
	**Overall**	**URTIs (J06.9)**	**Acute tonsillitis (J03.9)**	**Pneumonia (J18)**
Number of included physicians	327	310	53	24
Age				
<40 years	102 (31.19)	99 (31.94)	17 (32.08)	9 (37.50)
⩾40 & <60 years	207 (63.30)	193 (62.26)	34 (64.15)	15 (62.50)
⩾60 years	18 (5.50)	18 (5.81)	2 (3.77)	/
Gender				
Male	219 (66.97)	207 (66.77)	37 (69.81)	17 (70.83)
Female	108 (33.03)	103 (33.23)	16 (30.19)	7 (29.17)
Education [1]				
High school and below	55 (16.82)	55 (17.74)	1 (1.89)	2 (8.33)
College degree	120 (36.70)	112 (36.13)	24 (45.28)	11 (45.83)
Bachelor degree	152 (46.48)	143 (46.13)	28 (52.83)	11 (45.83)
Income (Yuan)				
<￥40,000	74 (22.63)	71 (22.90)	11 (20.75)	3 (12.50)
⩾￥40,000 & <￥80,000	138 (42.20)	130 (41.94)	24 (45.28)	16 (66.67)
⩾￥80,000 & <￥120,000	79 (24.16)	77 (24.84)	12 (22.64)	5 (20.83)
⩾￥120,000	36 (11.01)	32 (10.32)	6 (11.32)	/
Facility				
Urban healthcare centre of community	112 (34.25)	106 (34.19)	18 (33.96)	4 (16.67)
Rural township hospital	215 (65.75)	204 (65.81)	35 (66.04)	20 (83.33)
Department				
General practices	210 (64.22)	206 (66.45)	26 (49.06)	9 (37.50)
Internal medicine	75 (22.94)	67 (21.61)	18 (33.96)	9 (37.50)
Pediatrics	21 (6.42)	17 (5.48)	8 (15.09)	5 (20.83
Others	21 (6.42)	20 (6.45)	1 (1.89)	1 (4.17)
Professional title				
Doctor	159 (48.62)	153 (49.35)	21 (39.62)	12 (50.00)

Attending doctor	130 (39.76)	121 (39.03)	26 (49.06)	10 (41.67)
Associate professor/Professor	38 (11.62)	36 (11.61)	6 (11.32)	2 (8.33)
Clinical expertise				
<10 years	83 (25.38)	81 (26.13)	10 (18.87)	2 (8.33)
⩾10 & <20 years	102 (31.19)	94 (30.32)	18 (33.96)	14 (58.33)
⩾20 & <30 years	106 (32.42)	101 (32.58)	19 (35.85)	6 (25.00)
⩾30	36 (11.01)	34 (10.97)	6 (11.32)	2 (8.33)
Attending training regarding antibiotics				
Yes	267 (81.65)	250 (80.65)	45 (84.91)	22 (91.67)
No/Not clear	60 (18.35)	60 (19.35)	8 (15.09)	2 (8.33)

[1] Besides the bachelor program of medical college, college degree (leading to a vocational diploma) and high school and below (leading to a secondary vocational diploma) also existed due to the trade-off between quantity and quality. Physicians without bachelor's degrees are allowed to prescribe as assistant doctors in primacy cares.

Supplementary Table 2:

Interaction between physician tolerance of uncertainty and perceived patient tolerance of uncertainty on antibiotic use for outpatients with acute tonsillitis

**Different perceived patient tolerance of uncertainty [1]**	**Effect of physician tolerance of uncertainty**	
	**Marginal effect (95%CI) [2]**	**p value**
Perceived patient tolerance of uncertainty = 0	0.016 (0.007, 0.024)	<0.001
Perceived patient tolerance of uncertainty = 1	0.013 (0.006, 0.021)	<0.001
Perceived patient tolerance of uncertainty = 2	0.011 (0.004, 0.018)	0.001
Perceived patient tolerance of uncertainty = 3	0.009 (0.002, 0.015)	0.007
Perceived patient tolerance of uncertainty = 4	0.006 (0.000, 0.012)	0.046
Perceived patient tolerance of uncertainty = 5	0.004 (-0.003, 0.010)	0.245
Perceived patient tolerance of uncertainty = 6	0.001 (-0.006, 0.009)	0.702
Perceived patient tolerance of uncertainty = 7	-0.001 (-0.009, 0.007)	0.796
Perceived patient tolerance of uncertainty = 8	-0.004 (-0.013, 0.006)	0.453
Perceived patient tolerance of uncertainty = 9	-0.006 (-0.017, 0.005)	0.263
Perceived patient tolerance of uncertainty = 10	-0.009 (-0.021, 0.003)	0.162
Perceived patient tolerance of uncertainty = 11	-0.011 (-0.025, 0.002)	0.108
Perceived patient tolerance of uncertainty = 12	-0.014 (-0.029, 0.001)	0.076
Perceived patient tolerance of uncertainty = 13	-0.016 (-0.033, 0.001)	0.057
Perceived patient tolerance of uncertainty = 14	-0.019 (-0.038, 0.000)	0.045
Perceived patient tolerance of uncertainty = 15	-0.022 (-0.042, -0.001)	0.036
Perceived patient tolerance of uncertainty = 16	-0.024 (-0.046, -0.002)	0.030

[1] Patient tolerance of uncertainty was assessed based on physician estimation of patient ambiguity aversion, with higher scores indicates increasing patient intolerance of uncertainty;

[2] Marginal effect > 0 indicate higher chances to prescribe antibiotics.

Supplementary Data 1

Generation of Hubei primary care outpatient prescriptions dataset

The Hubei primary care outpatient prescriptions dataset is a dataset collecting routine clinical data regarding to outpatient healthcare services in primary cares in Hubei, including 89 of 2300 primary care facilities with 6.55 million citizens covered (11.05% of all populations in 2018).

The selection of primary care facilities applied two-stage cluster random sampling to ensure a representative sample. In the first stage, the provincial capital, Wuhan, and other 4 prefecture level city were randomly selected from the 16 cities in Hubei. In the next stage, an urban and a rural district were further randomly selected in each included city, consisting 5 urban districts and 5 rural districts, respectively. Within each district, all primary cares were included, covering 25 healthcare community centres in urban areas and 64 township hospitals in rural areas, respectively. The details of sampling process and results were showed in Figure S1:

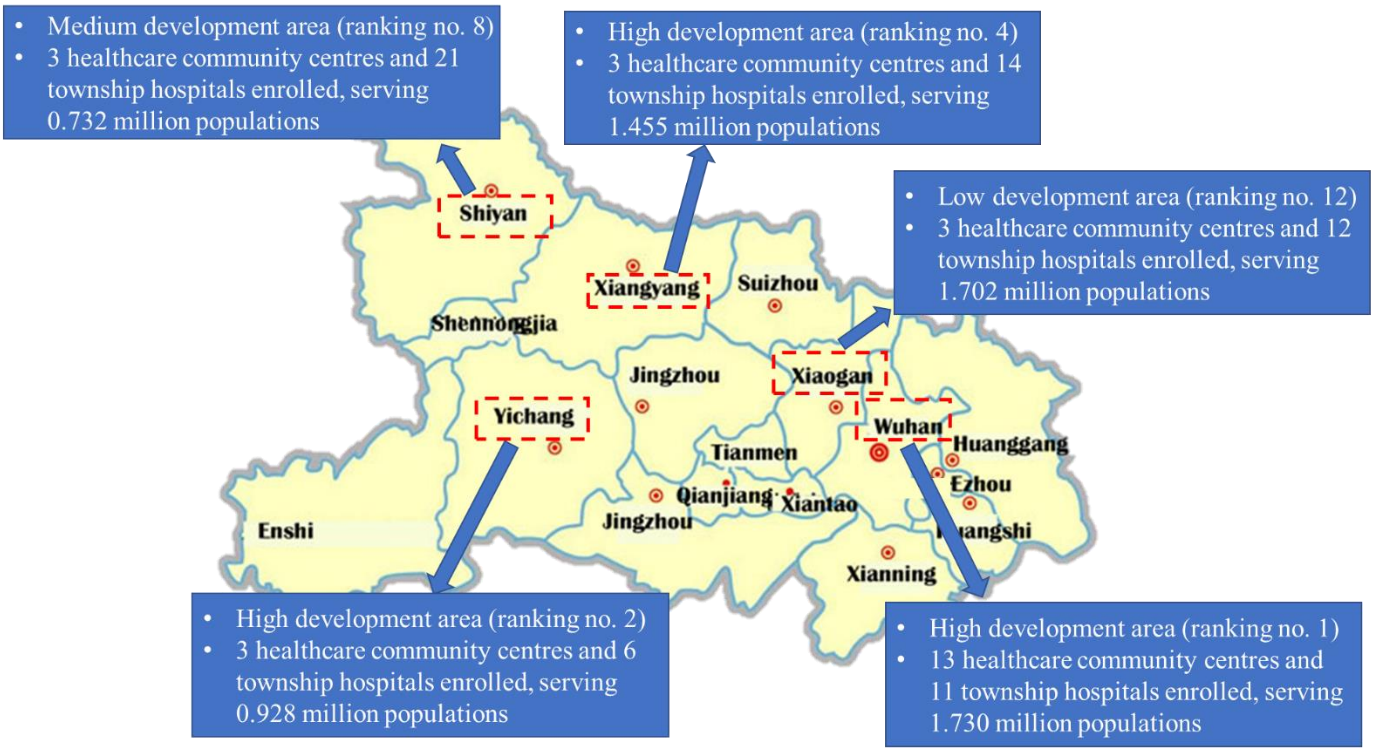

Figure S1 details of sampling process

Supplementary Data 2:

Survey Questionnaire (English translated version)

Survey of potential influencing factors of antibiotic use in primary cares

Dear physicians:

Greeting!

We are conducting a survey regarding to potential influencing factors of antibiotic use. We invited you to complete this 10-minute questionnaire and your responses are of great values to help us understand physicians' antibiotic prescribing behaviors. Your personal information would be confidential and the responses were only used for academic research only. Thanks very much for your participation.

School of Medicine and Health Management
Tongji Medical School, Wuhan

Part 1: Diagnostic uncertainty

Questions in this part **do not have a correct answer**, please fill in them according to your experience/attitude.

1. **In the past year for whom you prescribed them antibiotics, about what percentage that you were confident to give a clear diagnosis**

☐0% - 20%	☐20% - 40%	☐40% - 60%	☐60% - 80%	☐80% - 100%

2. **Please estimate the percentage of your patients who would have the following potential negative reactions to a discussion about a diagnostic uncertainty.**

	**0% - 20%**	**20% - 40%**	**40% - 60%**	**60% - 80%**	**80% - 100%**
2.1 Becoming unwilling to have the test or treatment	☐	☐	☐	☐	☐
2.2 Finding the information upsetting	☐	☐	☐	☐	☐
2.3 Becoming less trustful of medical experts	☐	☐	☐	☐	☐
2.4 Losing confidence in the test or treatment	☐	☐	☐	☐	☐

**3. To what extent you agree with the following statements**	Very disagree	Disagree	A little disagree	A little agree	Agree	Very agree
3.1 A good task is one in which what is to be done and how it is to be done are always clear	☐	☐	☐	☐	☐	☐
3.2 Before any important task, I must know how long it will take	☐	☐	☐	☐	☐	☐
3.3 I don't like to work on a problem unless there is a possibility of getting a clear-cut and unambiguous answer	☐	☐	☐	☐	☐	☐
3.4 The best part of working on a jigsaw puzzle is putting in that last piece	☐	☐	☐	☐	☐	☐
3.5 It really disturbs me when I am unable to follow another person's train of thought	☐	☐	☐	☐	☐	☐
3.6 If I am uncertain about the responsibilities involved in a particular task, I get very anxious	☐	☐	☐	☐	☐	☐
3.7 I am often uncomfortable with people unless I feel that I can understand their behavior	☐	☐	☐	☐	☐	☐

Part 2: Diagnostic ability test

Questions in this part **only have one correct answer**

1. **A girl, 8 years old, with fever and headache and muscle aches for 4 days. Physical examination: pharyngeal congestion, tonsillitis swollen at first degree, several of her classmates developed the disease. The most likely diagnosis is:**
   A. URTIs;
   B. Acute tonsillitis;
   C. Herpangina;
   D. Influenza;
   E. Kawasaki disease;
2. **A 20-year-old male, felt unwell after getting cold yesterday and developed cough and expectoration. Physical examination: the lungs were scattered with wet and dry rales. Blood routine: WBC 8.0 × 10^9/L, X-ray shows thickening of lung texture. The diagnosis should be considered first as:**
   A. Chronic bronchitis;
   B. Pneumococcal pneumonia;
   C. Mycoplasma pneumonia;
   D. Acute trachea-bronchitis;
   E. Tuberculosis;
3. **A patient, female, 16 years old. In early spring, she has a low fever, fatigue, dry cough, chest tightness for 10 days. Chest radiographs show thickened lung texture, blood routine: WBC 8.9×10^9/L, sputum culture (-). The most likely diagnosis is:**

A. Staphylococcus aureus;
B. Pneumococcal pneumonia;
C. Chlamydia pneumonia;
D. Infiltrating pulmonary tuberculosis;
E. Gram-negative bacilli pneumonia;

4. **A patient, female, 33 years old. She had fever, urinary frequency, urgency, dysuria, and low back pain for 3 days. Urine test revealed red blood cells, white blood cells, urine protein (+). The most likely diagnosis is:**
A. Acute nephritis;
B. Acute pyelonephritis;
C. Acute cystitis;
D. Acute urethritis;
E. Kidney tuberculosis;

5. **A patient, male, 7 months. Fever with diarrhea for 1 day with fever 39°C. Physical examination: pharyngeal redness with no signs of dehydration. Stool was egg drop soup, fat globules (+). The most likely cause of diarrhea is:**
A. Bacterial dysentery;
B. Overeating or improper feeding;
C. Viral enteritis;
D. Physiological diarrhea;
E. Pathogenic Escherichia coli enteritis;

Part 3: Demographics and working characteristics

1. Age	___________years
2. Gender	□Male □Female
3. Working place	□Community healthcare centers □Township hospitals
4. Medical specialization	____________
5. Professional title	□Doctor □Attending doctor □Associate chief doctor □Chief doctor
6. Education	□Secondary school and below □High school □College □Undergraduate □Postgraduate and above
7. Family annual income (¥)	□<20,000 □20,000 – 40,000 □40,000 – 60,000 □60,000 – 80,000 □80,000 – 100,000 □ 100,000 – 120,000 □120,000 – 140,000 □140,000 – 160,000 □160,000 – 180,000 □180,000 – 200,000 □> 200,000
8. Years of practice	___________years
9. Whether received training of antibiotics in the last year	□Yes □No/I don’t know
