# Supplementary material for: Does diagnostic uncertainty increase antibiotic prescribing in primary care?
Source: NPJ Prim Care Respir Med. 2021 Mar 25;31:17. doi: 10.1038/s41533-021-00229-9 (PMC7994848; doi:10.1038/s41533-021-00229-9)
Supplement: Supplementary file 2 — Reporting Summary [file 41533_2021_229_MOESM2_ESM.pdf]

nature research

Corresponding author(s): CHENXI LIU

Last updated by author(s): Feb 6, 2021

Reporting Summary

Nature Research wishes to improve the reproducibility of the work that we publish. This form provides structure for consistency and transparency in reporting. For further information on Nature Research policies, see our Editorial Policies and the Editorial Policy Checklist.

Statistics

For all statistical analyses, confirm that the following items are present in the figure legend, table legend, main text, or Methods section.

n/a	Confirmed	
☐	☒	The exact sample size (n) for each experimental group/condition, given as a discrete number and unit of measurement
☐	☒	A statement on whether measurements were taken from distinct samples or whether the same sample was measured repeatedly
☐	☒	The statistical test(s) used AND whether they are one- or two-sided<br>*Only common tests should be described solely by name; describe more complex techniques in the Methods section.*
☐	☒	A description of all covariates tested
☐	☒	A description of any assumptions or corrections, such as tests of normality and adjustment for multiple comparisons
☐	☒	A full description of the statistical parameters including central tendency (e.g. means) or other basic estimates (e.g. regression coefficient) AND variation (e.g. standard deviation) or associated estimates of uncertainty (e.g. confidence intervals)
☒	☐	For null hypothesis testing, the test statistic (e.g. F, t, r) with confidence intervals, effect sizes, degrees of freedom and P value noted<br>*Give P values as exact values whenever suitable.*
☒	☐	For Bayesian analysis, information on the choice of priors and Markov chain Monte Carlo settings
☐	☒	For hierarchical and complex designs, identification of the appropriate level for tests and full reporting of outcomes
☒	☐	Estimates of effect sizes (e.g. Cohen's d, Pearson's r), indicating how they were calculated

Our web collection on statistics for biologists contains articles on many of the points above.

Software and code

Policy information about availability of computer code

Data collection	No software was used.
Data analysis	All analyses were performed using STATA (version 14.0).

For manuscripts utilizing custom algorithms or software that are central to the research but not yet described in published literature, software must be made available to editors and reviewers. We strongly encourage code deposition in a community repository (e.g. GitHub). See the Nature Research guidelines for submitting code & software for further information.

Data

Policy information about availability of data

All manuscripts must include a data availability statement. This statement should provide the following information, where applicable:

- Accession codes, unique identifiers, or web links for publicly available datasets
- A list of figures that have associated raw data
- A description of any restrictions on data availability

The data of this study are derived from surveyed local institutions and restrictions apply to its availability, which were used under licence for the current study, and so are not publicly available. Data are however available from the authors upon reasonable request and with permission of surveyed local institutions and governments.

Field-specific reporting

Please select the one below that is the best fit for your research. If you are not sure, read the appropriate sections before making your selection.

☐ Life sciences ☒ Behavioural & social sciences ☐ Ecological, evolutionary & environmental sciences

For a reference copy of the document with all sections, see nature.com/documents/nr-reporting-summary-flat.pdf

Behavioural & social sciences study design

All studies must disclose on these points even when the disclosure is negative.

Study description	A quantitative cross-sectional study
Research sample	The participants were physicians in primary care in Hubei province, China.A two-stage cluster random sampling was adopted for the selection of primary care facilities in Hubei Province, China.<br>In the first stage, the provincial capital, Wuhan, and the other four prefecture-level cities were randomly selected from the 16 cities in Hubei. In the next stage, an urban and a rural district were further randomly selected in each included city, consisting of five urban districts and five rural districts, respectively. Within each district, all primary cares were included. This process resulted in 89 primary care facilities included (25 healthcare community centers in urban areas and 64 township hospitals in rural areas), which covered a geographic area with 6.55 million populations (about 11% of all populations in Hubei).<br>In total, 469 eligible physicians from selected facilities were invited to participate in this study. A total of 327 (69.72%) physicians returned a valid self-completed questionnaire. The majority of the physician respondents were male (66.97%) and at the age between 40 and 59 years (63.3%).
Sampling strategy	A two-stage cluster random sampling was adopted for the selection of primary care facilities in Hubei Province, China. In the first stage, the provincial capital, Wuhan, and the other four prefecture-level cities were randomly selected from the 16 cities in Hubei. In the next stage, an urban and a rural district were further randomly selected in each included city, consisting of five urban districts and five rural districts, respectively. Within each district, all primary cares were included. This process resulted in 89 primary care facilities included (25 healthcare community centers in urban areas and 64 township hospitals in rural areas), which covered a geographic area with 6.55 million populations (about 11% of all populations in Hubei).<br>In total, 469 eligible physicians from selected facilities were invited to participate in this study. A total of 327 (69.72%) physicians returned a valid self-completed questionnaire.
Data collection	Two steps were involved in data collection. The first step is a questionnaire survey of factors relevant to diagnostic uncertainty, collecting physician diagnostic ability, perceived frequency of diagnostic uncertainty, physician tolerance of uncertainty, and their perceived patient tolerance of uncertainty. The second step is an assessment of the antibiotic prescribing behaviors of these physicians based on their prescriptions in relation to the three selected illness conditions.<br><br>Questionnaire survey<br>Trained investigators were paired and deployed to the participating facilities to collect data over the period from November 2019 to January 2020. Before the survey, all investigators had completed a one-day intensive training, covering the background of the survey, detailed explanation of the survey instrument, and a simulated survey test. The pairing of investigators aimed to help each investigator when one omitted some required procedures to ensure a standardized survey process.<br><br>Extraction of prescriptions<br>Prescriptions issued by the 327 questionnaire respondents were extracted from the 2018 outpatient prescription dataset gathered by the local governments. The ICD-10 codes J06.9 (URTIs), J03.9 (acute tonsillitis), and J18 (pneumonia with unspecified organism) were used in identifying prescriptions for the three acute illness conditions. This resulted in a total of 207,804 prescriptions: 179,300 prescriptions for URTIs from 310 physicians; 21,347 prescriptions for acute tonsillitis from 53 physicians; and 7,157 prescriptions for pneumonia from 24 physicians.
Timing	From November 2019 to January 2020
Data exclusions	According to the recommendation from the World Health Organization for a reliable estimate of physician antibiotic prescribing patterns, physicians who issued at least 100 prescriptions for any one of the three selected diagnoses (ICD-10 codes J06.9, URTIs; J03.9, acute tonsillitis; and J18, pneumonia with unspecified organism) were eligible for the questionnaire survey.
Non-participation	142 physicians rejected to participate in this study or did not return a valid responses.
Randomization	Not applicable.

Reporting for specific materials, systems and methods

We require information from authors about some types of materials, experimental systems and methods used in many studies. Here, indicate whether each material, system or method listed is relevant to your study. If you are not sure if a list item applies to your research, read the appropriate section before selecting a response.

Materials & experimental systems

n/a	Involved in the study	
☒	☐	Antibodies
☒	☐	Eukaryotic cell lines
☒	☐	Palaeontology and archaeology
☒	☐	Animals and other organisms
☐	☒	Human research participants
☒	☐	Clinical data
☒	☐	Dual use research of concern

Methods

n/a	Involved in the study	
☒	☐	ChIP-seq
☒	☐	Flow cytometry
☒	☐	MRI-based neuroimaging

Human research participants

Policy information about studies involving human research participants

Population characteristics	The majority of the physician respondents were male (66.97%) and at the age between 40 and 59 years (63.3%). Nearly two thirds (64.22%) were general practitioners. Most respondents (74.62%) had over ten years of clinical experience. The vast majority (81.65%) reported attendance of antibiotic prescribing training.
Recruitment	A two-stage cluster random sampling was adopted for the selection of primary care facilities in Hubei Province, China. In the first stage, the provincial capital, Wuhan, and the other four prefecture-level cities were randomly selected from the 16 cities in Hubei. In the next stage, an urban and a rural district were further randomly selected in each included city, consisting of five urban districts and five rural districts, respectively. Within each district, all primary cares were included. This process resulted in 89 primary care facilities included (25 healthcare community centers in urban areas and 64 township hospitals in rural areas), which covered a geographic area with 6.55 million populations (about 11% of all populations in Hubei).<br><br>According to the recommendation from the World Health Organization for a reliable estimate of physician antibiotic prescribing patterns, physicians who issued at least 100 prescriptions for any one of the three selected diagnoses (ICD-10 codes J06.9, URTIs; J03.9, acute tonsillitis; and J18, pneumonia with unspecified organism) were eligible for the questionnaire survey. Based on the inclusion criteria, the sampling process generated a sample size of 469 eligible physicians from all included primary care facilities.<br><br>In total, 469 eligible physicians were invited to participate in this study. A total of 327 (69.72%) physicians returned a valid self-completed questionnaire.
Ethics oversight	This study has been approved by the Ethics Committee of Tongji Medical College, Huazhong University of Science and Technology (NO: 2020-S099). Written consent was obtained before the survey from each participant in the current study.

Note that full information on the approval of the study protocol must also be provided in the manuscript.
